# Apoptosis, autophagy and unfolded protein response pathways in Arbovirus replication and pathogenesis

**DOI:** 10.1017/erm.2015.19

**Published:** 2016-01-19

**Authors:** Mahmoud Iranpour, Adel Rezaei Moghadam, Mina Yazdi, Sudharsana R. Ande, Javad Alizadeh, Emilia Wiechec, Robbin Lindsay, Michael Drebot, Kevin M. Coombs, Saeid Ghavami

**Affiliations:** 1 Zoonotic Diseases and Special Pathogens, National Microbiology Laboratory, Public Health Agency of Canada, 1015 Arlington St., Winnipeg, Manitoba, Canada; 2 Young Researchers and Elite Club, Ardabil Branch, Islamic Azad University, Ardabil, Iran; 3 Faculty of Veterinary Medicine, University of Tehran, Tehran, Iran; 4 Department of Internal Medicine, College of Medicine, Faculty of Health Sciences, University of Manitoba, Winnipeg, Canada; 5 Department of Human Anatomy and Cell Science, College of Medicine, Faculty of Health Sciences, University of Manitoba, Winnipeg, Canada; 6 Department of Clinical and Experimental Medicine (IKE), Division of Otorhinolaryngology, Linkoping University, Linkoping, Sweden; 7 Department of Medical Microbiology and Infectious Diseases, College of Medicine, Faculty of Health Sciences, University of Manitoba, Winnipeg, Manitoba, Canada; 8 The Children Hospital Research Institute of Manitoba, Winnipeg, Canada

## Abstract

Arboviruses are pathogens that widely affect the health of people in different communities around the world. Recently, a few successful approaches toward production of effective vaccines against some of these pathogens have been developed, but treatment and prevention of the resulting diseases remain a major health and research concern. The arbovirus infection and replication processes are complex, and many factors are involved in their regulation. Apoptosis, autophagy and the unfolded protein response (UPR) are three mechanisms that are involved in pathogenesis of many viruses. In this review, we focus on the importance of these pathways in the arbovirus replication and infection processes. We provide a brief introduction on how apoptosis, autophagy and the UPR are initiated and regulated, and then discuss the involvement of these pathways in regulation of arbovirus pathogenesis.

## Introduction

Arthropod-borne viruses (commonly called arboviruses) typically circulate in nature through biological transmission among susceptible vertebrate hosts and blood-feeding arthropods such as mosquitoes (*Culicidae*), sand flies (*Psychodidae*), biting midges (*Ceratopogonidae*), black flies (*Simuliidae*) and ticks (*Ixodidae* and *Argasidae*) (Refs 1, 2). Most of the arboviruses that cause human diseases have RNA genomes and are within the families *Flaviviridae, Togaviridae, Bunyaviridae, Reoviridae* and *Rhabdoviridae* which, with few exceptions, are zoonoses that depend on wildlife or domestic animals for maintenance in nature (Ref. 1). Most of the arboviruses that cause disease in humans include: Alphaviruses (*Togaviridae: Alphavirus*), flaviviruses (*Flaviviridae: Flavivirus*), bunyaviruses (*Bunyaviridae*) and some viruses in the families *Reoviridae* and *Rhabdoviridae* (Refs 3, 4, 5, 6).

There are currently 534 viruses listed in the International Catalogue of Arboviruses, of which 214 are known to be, or are probably associated with arthropods, 287 viruses are reported to be possible arboviruses and 33 are considered to probably not be, or definitely not be, arboviruses. In total, 134 of the 534 arboviruses have been reported to cause illness in humans (Refs 7, 8).

**Figure 1.**
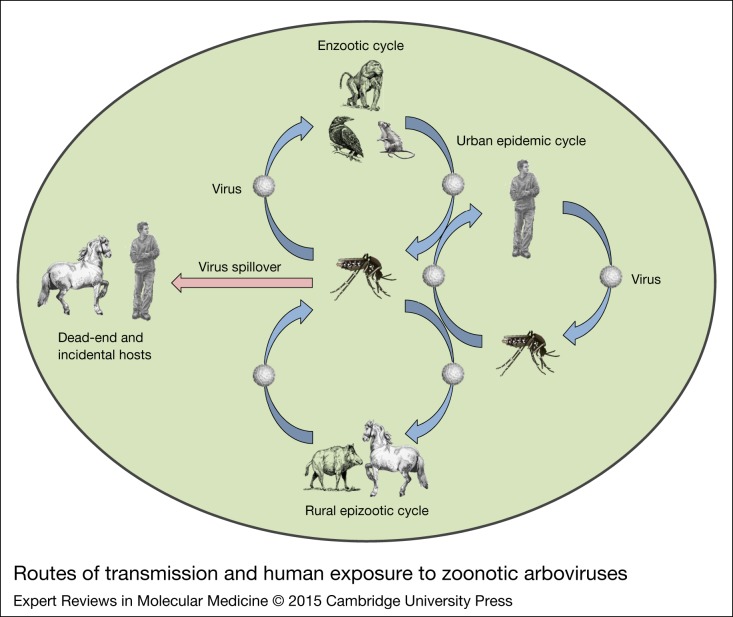
Routes of transmission and human exposure to zoonotic arboviruses. Infectious agents may be transmitted to humans by direct contact with infected animals, mechanical vectors or intermediate hosts. Arboviruses are maintained in mosquito-monkey, mosquito-rodent, mosquito-bird, mosquito-pig, mosquito-horse and mosquito-human cycles. The enzootic cycle occurs in the region where humans intrude into the natural foci of infection. The rural epizootic cycle is involved among domestic animals and mosquitos, and amplified in the presence of intermediate hosts, which result in representing a large reservoir of viruses and severe spillover effect to dead-end hosts. In urban settings, viruses are transmitted between humans and the mosquito vectors in an urban epidemic cycle, using humans for amplification (Ref. 10).

Arboviruses have a global distribution but the majority circulate in tropical areas where climatic conditions are favourable for year-round transmission. Arboviruses usually circulate within enzootic cycles involving wild or domestic animals with relatively few human infections (Ref. 9). Birds and rodents are the main reservoir hosts and mosquitoes and ticks are most often the vectors for the most important arboviruses (Table 1). ‘Spill-over’ of arboviruses from enzootic cycles to humans by enzootic or ‘bridge vectors’ can occur, under the appropriate ecological conditions. For most arboviruses, humans are dead-end or incidental hosts; however, there are several viruses such as *dengue, yellow fever* and *chikungunya* that primarily infect people during outbreaks and then begin to use humans as amplification sources (Ref. 9). Figure 1 illustrates the various mechanisms by which humans are infected by zoonotic and non-zoonotic arboviruses (Ref. 10).

**Table 1.** The lists of the most important arboviruses and their characterisation

Family	Virus/vector	Vertebrate host	Diseases in humans	Geographic distribution	Vaccine
Togaviridae	Chikungunya/mosquitoes (Refs 11, 12, 13)	Humans, Primates	SFI	Arica, Asia, Europe	NO
	Ross river/mosquitoes (Refs 13, 14)	Humans, Marsupials	SFI	Australia, South Pacific	NO
	Mayaro/mosquitoes (Refs 13, 15)	Birds	SFI	South America	NO
	O'nyong-nyong/mosquitoes (Ref. 13)	?	SFI	Africa	NO
	Sindbis/mosquitoes (Refs 11, 13)	Birds	SFI	Asia, Africa, Australia, Europe, USA	NO
	Barmah forest/mosquitoes (Ref. 14)	?	SFI	Australia	NO
	Eastern equine encephalitis/mosquitoes (Ref. 16)	Birds	SFI, ME	USA	YES
	Western equine encephalitis/mosquitoes (Ref. 17)	Birds, Rabbits	SFI, ME	USA	NO
	Venezuelan equine encephalitis/mosquitoes (Ref. 18)	Rodents	SFI, ME	USA	YES
Flaviviridae	Dengue 1-4/mosquitoes (Refs 19, 20)	Humans, Primates	SFI, HF	Tropical countries	NO
	Yellow fever/mosquitoes (Refs 19, 21, 22)	Humans, Primates	SFI, HF	Africa, South America	YES
	Japanese encephalitis/mosquitoes (Refs 19, 23)	Birds, Pigs	FSI, ME	Asia, Pacific, Australia	YES
	Murray valley encephalitis/mosquitoes (Refs 19, 24)	Birds	SFI, ME	Australia	NO
	Rocio encephalitis/mosquitoes (Ref. 19)	Birds	SFI, ME	South America	NO
	St. Louis encephalitis/mosquitoes (Refs 19, 25)	Birds	SFI, ME	Americas	NO
	West Nile/mosquitoes (Refs 19, 26)	Birds	SFI, ME	Africa, Asia, Europe, North America, Australia	NO
	Kyasanur forest disease/ticks (Refs 19, 27)	Primates, Rodents, Camels	SFI, HF, ME	India, Saudi Arabia	YES
	Omsk haemorrhagic fever/ticks (Refs 19, 28)	Rodents	SFI, HF	Asia	NO
	Tick-borne encephalitis/ticks (Refs 19, 29)	Birds, Rodents	SFI, ME	Europe, Asia, North America	YES
Bunyaviridae	Sandfly fever/sandflies (Ref. 30)	?	SFI	Europe, Africa, Asia	NO
	Rift valley fever/mosquitoes (Ref. 31)	?	SFI, HF, ME	Africa, Middle East	YES
	La Crosse encephalitis/mosquitoes (Ref. 32)	Rodents	SFI, ME	North America	NO
	California encephalitis/mosquitoes (Ref. 1)	Rodents	SFI, ME	North America, Europe, Asia	NO
	Congo-Crim. haemorrhagic encephalitis/ticks (Refs 20, 33)	Rodents	SFI, HF	Europe	YES
	Oropouche fever/midges (Refs 19, 34)	?	SFI	Central and South America	NO
Reoviridae	Colorado tick fever virus/ticks (Ref. 35)	Rodents	SFI	North America	No

HF, haemorrhagic fever; ME, meningoencephalitis; SFI, systematic febrile illness.

Arboviruses have been causing human disease for at least a thousand years but during recent decades some have newly emerged or re-emerged and a few have increased in importance because of human population expansion and increased urbanization, increased trade or travel and global climate change (Refs 2, 9, 36). Arthropod-borne viruses have been a serious public health concern, with viruses such as *dengue* (*DEN*) and *yellow fever* viruses causing millions of infections annually, while emerging arboviruses, such as *West Nile, Japanese encephalitis* (*JE*) and *Chikungunya* viruses (*CHIKV*) have significantly increased their geographical ranges in recent years (Refs 9, 37, 38, 39).

**Figure 2.**
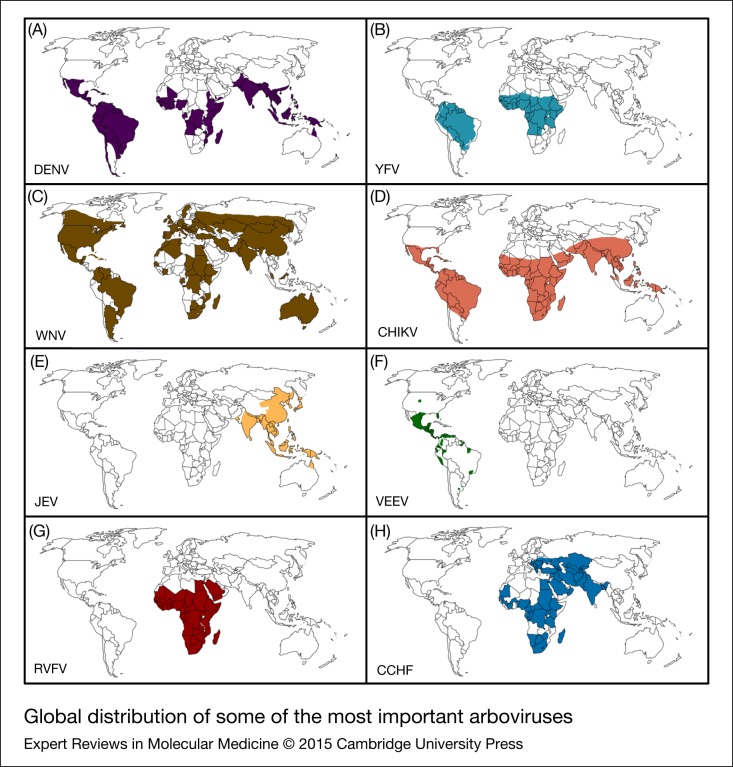
Global distribution of some of the most important arboviruses. (A) DENV, Dengue virus (Refs 41, 42), (B) YFV, Yellow fever (Refs 43, 44), (C) WNV, West Nile virus (Refs 45, 46, 47), (D) CHIKV, Chikungunya virus (Refs 47, 48), (E) JEV, Japanese encephalitis virus (Ref. 49), (F) VEEV, Venezuelan equine encephalitis virus (Ref. 47), (G) RVFV, Rift valley fever virus (Ref. 50), (H) CCHF, Crimean–Congo haemorrhagic fever (Ref. 51).

From a public health point of view, those arboviruses that produce viremia in humans and cause major mosquito-borne epidemics are most important (Ref. 40). Figure 2 shows world geographical distribution of the most important vector-born arboviruses. In the following section we will discuss some of the most common arbovirus-induced diseases.

## Common arbovirus-induced diseases

### Dengue/dengue haemorrhagic fever

The *dengue* viruses (*DENV*) are the only arboviruses that are fully adapted to the human host and its environment, thus eliminating the need for an enzootic transmission cycle (Refs 52, 53). Consequently, in recent years, transmission has increased in urban and semi-urban areas and has caused a major international public health concern (Refs 54, 55). *DEN* is now endemic in more than 100 countries in Africa, the USA, the Eastern Mediterranean, South-east Asia and the Western Pacific (Ref. 41). Severe *DEN*, previously known as Dengue haemorrhagic fever, occurs primarily in Asian and Latin American countries and has been a leading cause of hospitalization and death among children in these countries (Ref. 42). About 1.6 million cases of *DEN* were documented in the USA alone in 2010 (Ref. 42). The incidence of *DEN* has increased dramatically in recent years with over 2.5 billion people now at risk of contracting *DEN* (Ref. 56). It has been estimated that the annual number of *DENV* infections could be from 50 to 400 million cases with 25 000 deaths reported annually (Ref. 56).

Female *Aedes aegypti* mosquitoes can take blood meals from multiple human hosts during each feeding period, which increases the chance of infecting many human hosts (Ref. 42). *Aedes albopictus* acts as a secondary vector of *DENV* in Asia, and has recently expanded its geographical distribution both into and within parts of North America and Europe. Infection with *DENV* can be asymptomatic but often patients present with high fever, headache, pain behind the eyes, muscle and joint pains, nausea, vomiting, swollen glands or rash (Ref. 42). Severe DEN can potentially cause death because of plasma leakage, fluid accumulation, respiratory distress, severe bleeding or organ impairment (Ref. 42). There is no vaccine or treatment against this virus; therefore, environmental management, mosquito control and personal protection have been recommended (Ref. 56).

### Yellow fever

Yellow fever is a well-known disease that has caused major epidemics in the USA and Africa over the last four centuries (Ref. 1). It is endemic to parts of Africa and was introduced, along with its vector *Ae. aegypti*, into the Western Hemisphere in the early 1600s (Ref. 57). Globally over 900 million people are living in regions where Yellow fever is endemic and it is estimated that 200 000 cases of Yellow fever occur, resulting in 30 000 deaths each year (Ref. 43). There are no specific anti-viral treatments for Yellow fever, and the primary interventions are supportive care. Vaccination is the most important strategy to prevent Yellow fever. The current vaccine is highly effective and provides immunity within 30 days for 99% of vaccinated people (Refs 43, 44).

### West Nile virus

*West Nile* virus (*WNV*) was reported for the first time in Uganda in 1937 and then disappeared until the 1950s when it became widespread and caused disease outbreaks in the Middle East, India and Israel (Refs 1). *WNV* was recognized in the Western Hemisphere in the Northeastern USA in 1999 (Refs 52, 58, 59). In 2001, it became more widespread and 66 human cases with 9 deaths were reported from 10 states (Refs 44, 60, 61). In August 2001, *WNV* was identified in birds from Ontario, Canada (Ref. 62). The introduction of *WNV* into the USA has had a significant public health and economic impact. Millions of dollars have been spent on rebuilding and improving public health facilities to implement surveillance, prevention and control programs against *WNV* and other arboviral pathogens (Refs 63, 64). Currently, there is no human vaccine for *WNV* although several are available for horses (Ref. 52). Prevention and control is accomplished through effective surveillance coupled with targeted preventive measures and mosquito control (Ref. 1).

### Japanese encephalitis

*Japanese encephalitis* virus (*JEV*) is a Flavivirus that is maintained in an enzootic cycle involving *Culex* species of mosquitoes and aquatic birds (Refs 19, 23, 65). Pigs are efficient amplification hosts and their involvement greatly increases the risk of infection in humans (Ref. 65). Children are particularly susceptible to *JEV* infections and humans and horses are incidental hosts that can suffer a significant level of illness and death (Ref. 65). *JEV* is considered a leading cause of viral encephalitis worldwide, with more than 40  000 cases in Asia alone (Refs 66, 67). Climate, geography and host immune status play a significant role in *JEV* epidemiology (Refs 1, 68). *JEV* has been considered an emerging disease in the Indian subcontinent, parts of Southeast Asia and in the Pacific, and it caused a major epidemic in India for the first time in 1995 (Refs 69, 70, 71). JEV has also become a major public health problem in Nepal (Refs 72, 73, 74). It is possible that *JEV* has become established in northern Australia and possibly in other regions such as the USA where hosts and vectors are present (Ref. 47). Vaccination and changes in agricultural and animal husbandry practises are considered effective in controlling this arbovirus (Refs 52, 75).

### Rift valley fever

*Rift valley fever* virus (*RVFV*) has been responsible for numerous outbreaks of severe disease in domestic livestock (cattle, goats, camels and sheep) and humans over the past 70 years (Refs 76, 77). This virus was responsible for an outbreak affecting an estimated 200  000 people and devastated the sheep industry in Egypt from 1977 to 1979 (Refs 31, 78). It has now been reported in Saudi Arabia and Yemen (Refs 79, 80, 81), and recent outbreaks have occurred in Kenya, Tanzania and South Africa (Refs 82, 83). Concerns have been raised regarding the agricultural and medical impact that this zoonotic disease agent might have if it were to continue to expand its geographic range, either by natural means or intentional release (Refs 84, 85, 86, 87). Based on the outcome of the previous outbreaks, the threat from *RVFV* must not be underestimated as the consequences of this virus are dramatic, both for humans and livestock (Ref. 31).

### Venezuelan equine encephalitis

*Venezuelan equine encephalitis* (*VEE*) is an Alphavirus that has been isolated from a variety of animals including horses, rodents and mosquitoes (Refs 88, 89, 90). The geographic range of *VEE* virus is from Argentina to the USA. *VEE* virus includes five serotypes; two serotypes, AB and C, are considered epizootic and are pathogenic for horses (Refs 88, 89, 90), while the three serotypes D, E and F are considered to be enzootic. Both epizootic and enzootic variants of *VEE* virus cause a nonspecific viral syndrome in humans (Refs 89, 90). Epizootic virus infection can develop into encephalitis in a small number of cases. Death can occur following infection with either enzootic or epizootic serotypes of *VEE* virus (Ref. 1). *VEE* virus causes illness with symptoms similar to dengue and other mosquito-borne arboviruses; therefore, the numbers of reported cases may be an underestimate (Ref. 18). There is no treatment for this disease and also no licenced human vaccine for this virus except a live-attenuated vaccine for military forces and laboratory personnel (Ref. 91).

## Viruses and autophagy, apoptosis and unfolded protein response (UPR)

Many viruses hijack host cell responses for their own benefit and use them as complementary mechanisms for replication and infection. Some of the most important host mechanisms that are usually affected by viral infection are pathways involved in cell death and cellular responses against environmental stress. These mechanisms include apoptosis (i.e. programmed cell death I), autophagy (programmed cell death II) and UPR. These mechanisms play essential functions in regulating cell fate and are important for normal cellular functions. In addition, these mechanisms are tightly regulated and can affect each other. They are usually interconnected and also ‘cross-talk’ with each other. We will briefly review the general concepts of apoptosis, autophagy and UPR and explain their cross-talk and regulatory mechanisms. We will then focus on the role of apoptosis, autophagy and UPR in arbovirus replication and infection and then describe different possible therapeutic approaches for arboviruses by discussing the involvement of apoptosis, autophagy and how they may determine therapeutic strategies.

## An overview of autophagy, apoptosis and UPR

### Autophagy

Lysosomes are the final destination for degradation of long-lived and dysfunctional cellular components through autophagy, a highly regulated catabolic process. This process is essential for maintaining cellular integrity, homeostasis, survival, differentiation and development (Refs 92, 93, 94, 95). In mammals, the role of nutrient deprivation, hormonal stimuli, including glucagon and insulin, and other autophagy activation cues such as temperature, oxygen concentration and cell density have been elucidated (Refs 96, 97, 98, 99). There are three different types of autophagy, all of which differ in their mechanisms and functions: chaperone-mediated autophagy (CMA), microautophagy and macroautophagy (Refs 100, 101, 102, 103, 104, 105). During CMA, specific cytosolic proteins are selectively tagged by the CMA substrate chaperone complex and then moved to the lysosome for degradation (Refs 104, 106, 107). This is the only form of autophagy in which no vesicular traffic is involved (Ref. 108). Microautophagy directly targets small proteins and organelles using lysosomes (Refs 109, 110, 111). However, macroautophagy is the major regulated catabolic mechanism by which the bulk of damaged cytoplasmic proteins and organelles are sequestered within an autophagosome (Refs 112, 113, 114). In this review, we will focus on macroautophagy (referred to herein as ‘autophagy’).

**Figure 3.**
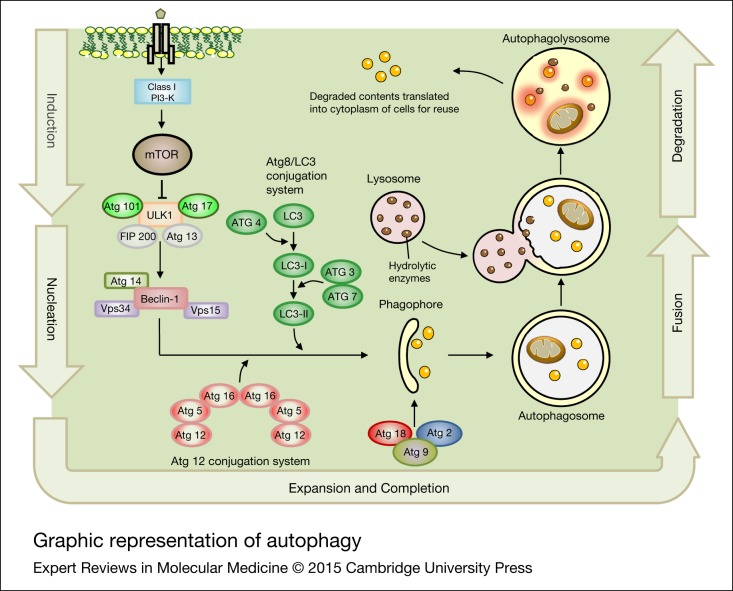
Graphic representation of autophagy. Autophagy is a process for the degradation and recycling of damaged or unnecessary cellular compartments, which has several tightly regulated steps including induction, nucleation, expansion and completion, fusion and degradation. The mTOR is known as the key regulator of autophagy induction and can be suppressed by ULK1, leading to trigger VPS34-Beclin 1-class III PI3-kinase complex. Several different membrane pools contribute to the formation of the phagophore. The Atg proteins (Atg2, Atg9, Atg18) are essential for phagophore formation. The ATG and LC3 conjugation system also contribute in autophagosome membrane formation and elongation. The autophagolysosome then is formed by fusion of the autophagosome with a lysosome to degrade and reuse the compounds. ATG, autophagy-related genes; mTOR, mammalian target of rapamycin.

The first step in autophagy (see Fig. 3) involves formation and expansion of a double-membrane structure, which is called the ‘isolation membrane’ or ‘phagophore’. The edges of this membrane eventually fuse to form a new double membrane-bound vacuole, known as the autophagosome that sequesters the cytoplasmic cargo. The autophagolysosome is formed by fusion of the autophagosome with a lysosome and lysosomal contents are degraded by hydrolytic enzymes (Refs 115, 116, 117, 118). As a result of degradation, nucleotides, amino acids and free fatty acids (FFAs) are generated and then reused for energy metabolism, macromolecular production and biosynthesis (Refs 119, 120). It is assumed that the different steps in macroautophagy are mediated by autophagy-related genes (ATG), which encode proteins involved in autophagy (Refs 121, 122). These proteins have been classified into five different functional categories: (i) a protein serine/threonine kinase complex that responds to upstream events such as target of rapamycin (TOR) kinase (Atg1/ULK1, Atg13 and Atg17); (ii) a lipid kinase group that controls vesicle nucleation (Atg6/Beclin1, Atg14, Vps34/PI3KC3 and Vps15); (iii) two ubiquitin-like conjugation pathways that stimulate vesicle expansion (the Atg8 and Atg12 conjugation systems); (iv) a recycling pathway that is required for disassembly of Atg proteins (Atg2, Atg9, Atg18); and (v) vacuolar permeases that permit the efflux of amino acids from the degradative compartment (Atg22) (Refs 93, 119). The mammalian TOR (mTOR) kinase acts as a negative regulator of autophagy and is a central controller of cell growth, aging and proliferation (Refs 123, 124). Under starvation conditions, inhibited mTOR induces autophagy through phosphorylation of the Ulk1-Atg13-FIP200-Atg101 complex (Refs 125, 126), leading to localization of Ulk1/2 and Atg13 to the autophagic isolation membrane (Refs 127, 128). During the initiation step of autophagy, Beclin 1 interacts with Vps34, which contributes to Atg protein recruitment and autophagosome nucleation (Refs 129, 130). Interaction with various Beclin-1-interacting proteins facilitates the coordination of these events (Ref. 131). LC3, the mammalian ortholog of Atg8, is cleaved by Atg4 and then conjugated to the polar head of phosphatidylethanolamine (PE) to generate LC3-II, which is necessary during the elongation step of autophagy (Refs 132, 133). Hence, the autophagosome is regulated in response to the Beclin-1/Vps34/UVRAG complex, known as the maturation step (Refs 134, 135, 136). An overview of autophagy is summarised in Figure 3.

### Apoptosis

**Figure 4.**
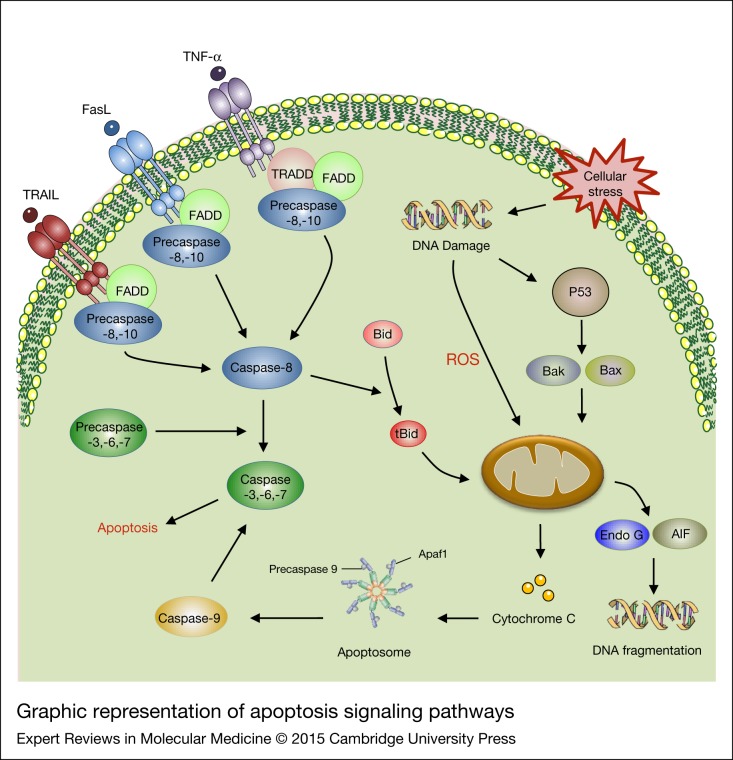
Graphic representation of apoptosis signalling pathways. Apoptosis is initiated via two different routes including extrinsic and intrinsic apoptotic pathways. The extrinsic signals are initiated by cell death ligands (e.g. FasL, APO-2L, TRAIL, TNF) and activate FADD and subsequently cleave pro-caspase-8. Cleavage of pro-caspases-8 and -10 initiate activation of caspases-8 and -10, which later can directly trigger effector caspases including caspases-3, -6 and -7. The intrinsic pathway is stimulated via DNA damage. Once DNA damage occurs, p53 is activated and induces apoptosis in a mitochondria-dependent manner. In this pathway, pro-apoptotic and antiapoptotic proteins are up- and down-regulated, leading to release of cytochrome c. Released cytochrome c later can activate caspase 9 which in turn activates caspase-3. FasL, Fas (Apo-1/CD95) ligand; TNF, tumour necrosis factor receptor TRAIL, TNF, tumour necrosis factor receptor.

There are two main functionally distinct pathways for apoptosis induction (Fig. 4): the extrinsic and the intrinsic mitochondrial pathways (Refs 137, 138, 139). Caspases are involved in most of the apoptotic processes and are activated by ligation of death receptors [tumour necrosis factor receptor (TNFR), Fas, TNF-related apoptosis-inducing ligand (TRAIL)] or release of specific proteins from the mitochondria (Refs 140, 141). However, accumulating evidence suggests that the two pathways are intimately intertwined (Refs 138, 142), which will be described in the next sections. The extrinsic apoptosis cascade is stimulated after the binding of cell surface receptors to their ligands, resulting in Fas-associated protein with death domain (FADD)-dependent activation of initiator caspases, namely caspase-8, and subsequently caspase-3 and -7 (Refs 143, 144). As a consequence, effector caspases (i.e. caspase-3 and caspase-7) are dimerized and activated and, once active they can cause apoptosis (Refs 141, 145).

The mitochondrial apoptotic death mechanism integrates various extracellular stimuli including drugs, nutrients and radiation and also different intracellular stimuli such as oxidative stress, oncogene expression, endoplasmic reticulum (ER) stress and DNA damage (Refs 146, 147). The apoptotic signals in this pathway converge on the mitochondria to release apoptogenic proteins such as cytochrome c, apoptosis-inducing factor (AIF), Smac/DIABLO, Omi/HtrA2 and mitochondrial endonuclease G (Refs 148, 149, 150, 151). The Bcl-2 family of proteins serve as important regulators of the release of these mitochondrial proteins that can be divided into two classes: (i) antiapoptotic members (e.g. Bcl-2 and Bcl-xL); and (ii) proapoptotic members (e.g. Bax, Bak, Bid, Bad, Noxa, Puma and others) (Refs 152, 153). Up-regulation of proapoptotic proteins or down-regulation of antiapoptotic proteins can cause an increase in permeability of the mitochondrial membrane, which later promotes release of cytochrome c and other proteins into the cytosol (Refs 151, 154, 155, 156). In the presence of deoxyadenosine triphosphate (dATP), the released cytochrome c interacts with Apaf-1 and caspase-9 and forms a ternary complex, leading to activation of caspase-3 and then apoptosis (Refs 142, 157, 158). In addition, p53 plays a stimulating role in intrinsic apoptosis induction (Refs 159, 160, 161). Thus, the two direct p53 transcriptional targets Noxa and Puma can mediate the pro-apoptotic activity of Bax and Bak, and thereby promote apoptosis (Refs 162, 163).

It is widely accepted that there is cross-talk between the two extrinsic and intrinsic pathways, such that activity in one pathway interferes with signalling steps in the other pathway (Ref. 141).

The pro-apoptotic cytochrome c-releasing factor Bid is positioned to serve as a link between the extrinsic death receptor pathway and the intrinsic pathway (Ref. 154). Cleavage of the BID protein in the cytoplasm by caspase-8 causes Bid to localise in the cytosol while truncated Bid translocates to the mitochondria and activates the mitochondrial pathway after apoptosis induction through death receptors, and can be used to amplify the apoptotic signal (Ref. 164). Although Bid is a downstream target of caspase-8 in the extrinsic apoptotic pathway, it also acts as ligand for Bax and Bak, causing caspase-9 activation (Refs 154, 165). Caspase-9 activation proteolytically activates downstream caspases (e.g. caspases-3,-6,-7), which, in turn, can result in apoptosis (Refs 166, 167).

### UPR

**Figure 5.**
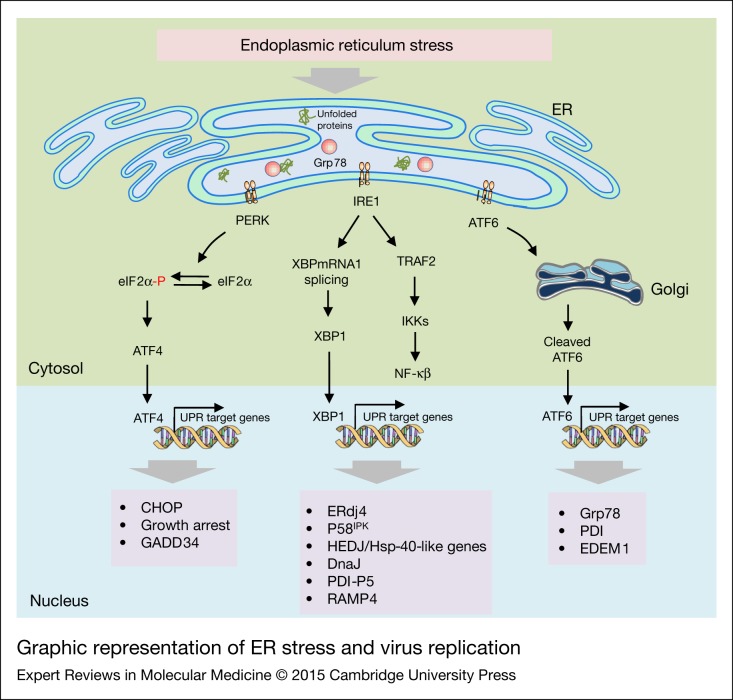
Graphic representation of ER stress and virus replication. ER stress is enhanced in the viral infected cells and activates UPR proteins (e.g. PERK, ATF6, and IRE1). Activated PERK leads to induce ATF4 via phosphorylation of eIF2α, causing attenuation of translation and inducing genes encoding CHOP. Upon IRE1 activation, TRAF2 and sXBPmRNA1 splicing are initiated in the cytoplasm, subsequently leading to activation of UPR target genes. The degradation of ATF6 is increased through recruitment of ATF6, a UPR sensor. ATF6 translocates to the Golgi and is cleaved to a nucleus targeting form that promotes expression of UPR-responsive genes. The consequences of UPR activation are necessary for viral replication and pathogenesis. ATF, activating transcription factor; CHOP, C/EBP homologous protein; ER, endoplasmic reticulum; IRE1, inositol-requiring enzyme; PERK, protein kinase RNA like ER kinase; UPR, unfolded protein response.

The ER contains an extensive network of tubules, sacs and cisternae, which extend from the cell plasma membrane through the cytoplasm and to the nuclear envelop through a continuous connected network (Refs 168, 169). The ER is the main sub-cellular compartment involved in proper folding of proteins and their maturation. Approximately one-third of the total proteins are synthesised in the ER. Many different perturbations can alter the function of the ER leading to unfolding or misfolding of proteins in the ER. This condition is referred to as ER stress (Refs 169, 170). The ER creates a series of adaptive mechanisms to prevent cell death complications and these together are referred to as the UPR (Refs 170, 171). The UPR can be involved in the secretory pathway leading to restoration of protein folding homeostasis. However, if there is too much stress on the ER, and the ER cannot cope with this stress, it will eventually lead to cell death (Ref. 172). The UPR also plays an important role in maintaining cellular homeostasis of specialised secretory cells such as pancreatic beta cells, salivary glands and plasma B cells (Ref. 170). It is becoming increasingly evident from animal models that UPR has several functions that are not directly linked to protein folding including inflammation, energy control and lipid and cholesterol metabolism (Ref. 170). The existence of UPR was first reported by Kozatsumi et al. more than 25 years ago (Ref. 173). They showed that glucose regulated proteins (GRPs) that are associated with the ER are up-regulated upon sensing the presence of unfolded or misfolded proteins in the ER (Ref. 173). While the mechanisms and signalling events behind it were not known at the time, today we have a much better understanding of the UPR and how these events are regulated in the ER at the molecular level. ER stress response signals are constantly monitored by three main classes of sensors. These include inositol requiring enzyme 1 alpha (IRE-1α) and IRE-1β, protein kinase RNA like ER kinase (PERK) and activating transcription factor 6 (ATF6; both α and β isoforms) (Fig. 5). In normal healthy cells these sensors are in an inactive state.

#### IRE1

This is a type I transmembrane protein receptor having an N-terminal ER luminal-sensing domain. The cytoplasmic C-terminal region contains both an endoribonuclease domain and a Ser/Thr kinase domain (Ref. 169). There are two homologues of IRE1: IRE1α and IRE1β. Activation of IRE1 involves dissociation from Grp78, followed by dimerization, oligomerization and trans-autophosphorylation, which leads to conformational changes and activation of its RNase domain (Ref. 170). Activated IRE1 excises a 26-nucleotide intron region from mRNA that encodes the transcription factor X-box binding protein 1 (XBP1). Dissociation of this 26-nucleotide intron region from XBP1 leads to a shift in the coding reading frame and produces a more stable form of XBP1 called XBP1 spliced form (XBP1s) (Ref. 170). IRE1-XBP1s signalling axis modulates pro-survival responses by targeting many genes involved in protein folding, maturation and ER-associated degradation (Ref. 169). XBP1 also modulates phospholipid synthesis which is required for ER expansion under ER stress (Ref. 174). Some examples of XBP1 target genes include ERdj4, P58^IPK^, human ER-associated DNAJ (HEDJ), DnaJ/Hsp-40-like genes and protein disulphide isomerase (PDI) P5 (PDI-P5) and ribosome associated membrane protein 4 (RAMP4) (Ref. 169). Different studies have shown that activation of IRE1 signalling is robust at first but as time progresses it diminishes (Refs 169, 175). However, artificial maintenance of IRE1 signalling is achieved by a chemically-activated mutant form of IRE1, which is positively correlated with enhanced cell survival conditions under ER stress, suggesting that IRE1 signalling mainly plays a role in a pro-survival pathway (Refs 169, 175, 176).

#### ATF6

ATF6 is a type II transmembrane protein that contains a basic leucine zipper (bZIP) transcription factor domain in its cytosolic terminus (Refs 169, 177). The ATF6 family of ER transducers include ATF6 α, ATF6 β, old astrocyte specifically induced substance (OASIS), LUMAN (also called CREB3), BBF2 human homolog on chromosome 7 (BBF2H7), cyclic-AMP responsive element binding protein hepatocyte (CREBH) and CREBP4 (Ref. 174). Unlike IRE1, ATF6 does not undergo oligomerization, dimerization and autophosphorylation. Under ER stress conditions, Grp78 dissociate from ATF6 thus uncovering the Golgi localisation signal of ATF6. Activated ATF6 translocates into the Golgi complex where it undergoes cleavage by site-1 and site-2 proteases (Ref. 177). Thus, the ATF6 N-terminal cleavage product translocates to the nucleus and regulates the expression of genes that are associated with the ER-associated protein degradation pathway. Some of the ATF6 target genes include Grp78, PDI and ER-degradation enhancing-a-mannosidase-like protein 1 (EDEM1). All these proteins work closely to reduce unfolded proteins in the ER lumen (Ref. 169). ATF6 also activates pro-survival transcription factor and IRE1 target gene XBP1 (Refs 178, 179). Similar to that of IRE1 signalling, ATF6 is activated by the UPR but is not sustained throughout the UPR response. ATF6 signalling is primarily for pro-survival but in some cases, ATF6 signalling activates the pro-apoptotic transcription factor C/EBP homologous protein (CHOP) during prolonged ER stress (Ref. 178).

#### PERK

This is a type I ER transmembrane protein having an ER luminar sensor domain and a cytoplasmic domain. The cytoplasmic domain contains Ser/Thr kinase activity. Upon activation by UPR, PERK dissociates itself from Grp78 and undergoes dimerization and trans-auto phosphorylation (Refs 169, 172, 180). Activated PERK phosphorylates eukaryotic translation initiation factor 2α (eIF2α). PERK-mediated phosphorylation of Ser51 in eIF2α reduces the activity of eIF2α complex and leads to the inhibition of protein synthesis. This rapidly reduces the number of proteins entering the ER and this can lead to a pro-survival effect on the cell (Refs 170, 172, 181). Phosphorylation of eIF2α also allows translation of mRNAs containing short open reading frames in their 5′ UTR regions. Such translated proteins include activating transcription factor 4 (ATF4) (Ref. 170). ATF4 controls expression of many proteins involved in redox processes and amino acid metabolism, and it modulates the expression of ER chaperones and foldases (Ref. 170). ATF4 also regulates important genes involved in ER apoptosis such as CHOP and growth arrest and DNA damage inducible 34 (GADD34) (Ref. 170). GADD34 is involved in a feedback loop to dephosphorylate eIF2α by protein phosphatase IC (PPIC) to restore protein synthesis (Refs 170, 182). Another substrate for activated PERK is nuclear factor (erythroid-derived 2 factor)-related factor (Nrf2). In normal cells, Nrf2 is present in the cytoplasm in association with cytoskeletal anchor kelch-like Ech-associated protein (KEAP1). Upon activation PERK phosphorylates Nrf2 and this helps Nrf2 to dissociate from KEAP1 and translocate into the nucleus (Refs 169, 183). Upon translocation into the nucleus Nrf2 induces the expression of genes that have an anti-oxidant response element (ARE) within their promoter such as heme oxygenase 1 (HO-1), aiding in protein folding and helping to restore ER homeostasis (Refs 169, 183). The role of Nrf2 as a pro-survival factor is further shown by the fact that cells devoid of Nrf2 display increased sensitivity to cell death via apoptosis after ER stress (Refs 169, 183). The overall UPR signalling pathway is shown in Figure 5.

## The role of autophagy in arbovirus replication

**Figure 6.**
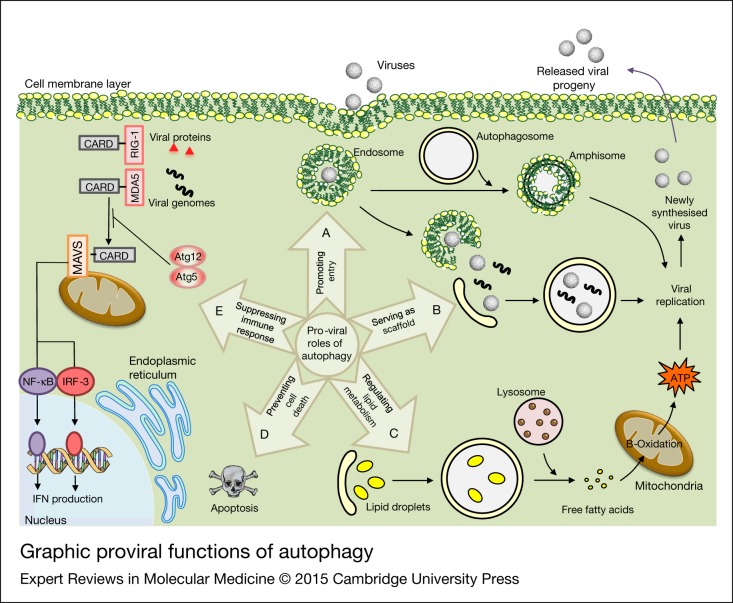
Graphic proviral functions of autophagy. There are five possible mechanisms for modulating viral replication by autophagy. Amphisome formation is thought to be beneficial for viral cellular entry and replication. Induction of autophagosome formation is also important for some virus’ replication. Furthermore, viruses initiate autophagy to benefit from lipid droplets as an energy source during viral replication. Free fatty acids are liberated from lipid droplets during autophagy to produce ATP. Viruses also stimulate autophagy to subvert immune responses by selectively degrading key regulatory molecules. Another mechanism is that viruses promote their replication by prolonging cell survival and suppressing cell death. The mechanistic details related to proviral functions of autophagy are discussed in the text.

Although autophagy was initially proposed as a physiological cellular response to environmental stress followed by virus amplification, increasing evidence now indicates that several viruses may use autophagy as a survival strategy to support their life cycle, which is known as ‘pro-viral autophagy’ (Refs 131, 138, 184, 185) (Fig. 6). Virus-induced induction of autophagy seems to be associated with replication/translation of many arboviruses like *DENV, JEV, CHIKV*, rotavirus, and *epizootic haemorrhagic disease* virus (*EHDV*, an orbivirus) (Refs 186, 187, 188, 189, 190, 191). The results that were obtained by monitoring LC3 lipidation in *JEV*-infected NT-2 cells, a pluripotent human testicular embryonal carcinoma cell line treated with Rapamycin and 3-methyladenine, revealed that there was a direct relationship between autophagy and viral replication The results were confirmed using an Atg5/Beclin-1 knock down model (Ref. 187). Most commonly, in many eukaryotic cells, it is apparent that the initiation of autophagy can be enhanced in infected *DENV* cells; in addition, the replication of *DENV* is positively linked to autophagy induction (Ref. 192). However, *DENV* viral replication has been shown to be limited in monocytes, which suggests a possible cell-specific relationship between activated autophagy and *DENV* production (Ref. 193). *WNV* induces autophagy even though its replication is autophagy independent (Ref. 194). The importance of virus-induced autophagy and up-regulation of viral replication has also been shown in *CHIKV*-infected cells (Ref. 188). The Orbivirus *EHDV* induces autophagy, apoptosis and c-Jun N-terminal kinase (JNK) activation, and phosphorylates c-Jun, all of which seem to benefit viral replication (Ref. 190). *JEV* also induces autophagy in the early stage of infection and the inoculated viral particles traffic to autophagosomes for subsequent steps of viral infection (Ref. 187). In vivo studies showed that autophagy played a supporting role in *DENV-2* replication and pathogenesis (Ref. 195).

Although the function or functions of autophagy in promoting virus replication are not completely understood, experimental evidence suggests that there are multiple autophagy pro-viral mechanisms, including serving as a scaffold for viral replication, contributing to viral entry, regulation of lipid metabolism, suppressing innate immune responses and preventing cell death (Ref. 196). A group of arboviruses including *DENV*, and *JEV* may need to invoke autophagy components such as the autophagosome, amphisome and autolysosome to: (i) serve as a scaffold for viral replication; and (ii) escape from the immune system (Refs 187, 197, 198, 199). The amphisomes play major roles in *DENV* entry and localisation of viral translation/replication constituents (Ref. 199). *DENV-2* needs pre-lysosomal fusion vacuoles (amphisomes) while *DENV-3* interacts with both amphisomes and autophagolysosomes as the sites for their viral translation/replication complexes (composed of viral RNA and proteins) (Ref. 199). *Poliovirus* and *CHIKV* also stimulate autophagosome formation as a site for aggregation of viral translation/replication complexes (Refs 188, 189, 200). After *DENV* and *JEV* induce autophagy, the presence of viral replication/translation complexes in both the autophagosome and the endosome suggests an auxiliary role for autophagosome–endosome fusion in viral entry (Refs 187, 201). Autophagy can regulate lipid metabolism (lipophagy) through modulating the degradation of triglycerides that have accumulated in cytosolic lipid droplets (Ref. 202). Lipid droplet usage as an energy source is another autophagy-mediated pro-viral mechanism that is used for *DENV* replication (Ref. 203). Thus, lipid droplets are sequestered in autophagosomes and delivered to lysosomes for degradation to generate FFAs from triglycerides (Ref. 203). The released FFAs are imported to mitochondria and they undergo β-oxidation to produce ATP for viral replication (Ref. 203).

## The innate antiviral immune response

The innate antiviral response is initiated by binding of pattern recognition receptors (PRRs), retinoic acid-inducible gene (RIG) and Melanoma differentiation-associated protein 5 (MDA5) to intracellular viral pathogen associated molecular patterns (PAMPs) (Ref. 204). The interaction of PRR-PAMP with mitochondrial antiviral-signaling protein (MAVS) through Caspase activation and recruitment domain (CARD)–CARD homotypic reaction leads to signalling cascades that ultimately activate nuclear factor-κB (NF-κB) and interferon regulatory factors (IRF-3) (Refs 205, 206). Inhibiting interferon (IFN) production followed directly from interaction of Atg5-Atg12 with the CARD of RIG, and MDA5 can promote vesicular stomatitis virus (VSV) replication (Ref. 207). Although the exact mechanism of autophagosome accumulation in *JEV* replication is still unclear, several studies have demonstrated the importance of fusion between autophagosomes and lysosomes and also autophagy in reducing MAVS-IRF3 activation to facilitate virus replication (Ref. 208). Additionally, it has been suggested that autophagy promotes cell survival by delivering damaged mitochondria to lysosomes during *JEV* infection (Ref. 208).

Optimal Flavivirus (e.g. *DENV2*) replication/translation is associated with the nonstructural viral protein NS4A in up-regulating PI3-K-dependent autophagy, and preventing cell death (Ref. 209). Recently, NS4A has been characterised as a main component of the membrane-bound *DENV2* replication complexes (Ref. 210). With attention to the cross-talk between autophagy and apoptosis, it is becoming apparent that autophagy postpones apoptosis and promotes *CHIKV* propagation by inducing the IRE1α–XBP-1 pathway in conjunction with ROS-mediated mTOR inhibition (Ref. 211). A schematic representation of autophagy and arbovirus replication is summarised in Figure 6.

## The role of apoptosis in arbovirus replication

**Figure 7.**
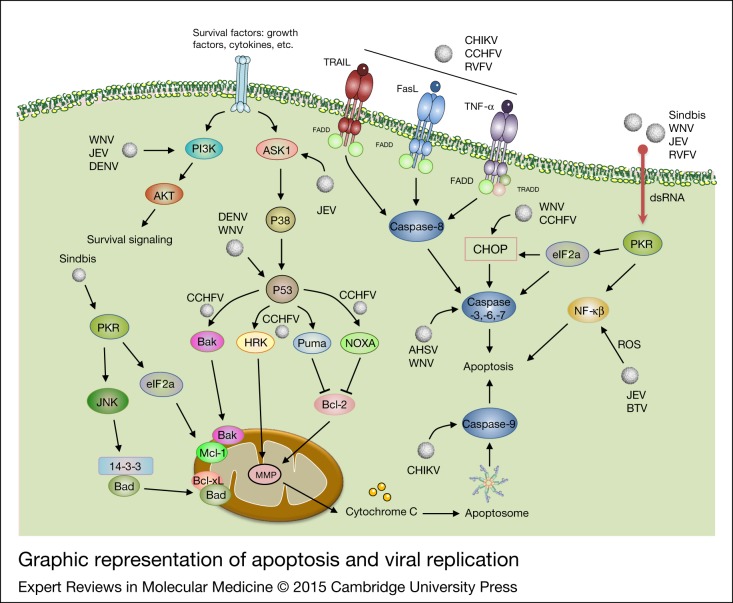
Graphic representation of apoptosis and viral replication. Viral infection, in general, can induce both intrinsic and extrinsic apoptotic pathways. Viruses like CHIKV, CCHFV and RVFV initiate extrinsic signals through cell death ligands (e.g. FasL, APO-2L, TRAIL, TNF), causing caspases-8 activation which then triggers caspases-3, -6 and -7). AHSV and WNV directly trigger caspase 3; however, CHIKV targets caspase 9. DENV and WNV affect the intrinsic pathway of apoptosis through stimulation of P53. Once P53 is activated, mitochondria-dependent apoptosis can be activated. Viral infection can also induce PKR and this kinase can affect eIF2a, resulting in activation of effector caspases and initiation of apoptosis. Viruses can also have anti-apoptotic activity. DENV, WNV and JEV trigger survival signalling through PI3K-AKT signalling pathway. PKR can be initiated by Sindbis virus which leads to inhibition of cellular translation through eIF2a phosphorylation, suppressing Mcl-1 biosynthesis. Sindbis virus can regulate 14-3-3 through activation of JNK followed by induction of PKR (for other details see text). AHSV, African horse sickness virus; CHIKV, Chikungunya virus; CCHF, Crimean–Congo haemorrhagic fever virus; DENV, Dengue virus; FasL, Fas (Apo-1/CD95) ligand; JEV, Japanese encephalitis virus; JNK, c-Jun N-terminal kinases; TNF, tumour necrosis factor receptor; TRAIL, TNF-related apoptosis-inducing ligand; PKR, (dsRNA)-activated protein kinase; RVFV, Rift valley fever virus; WNV, West Nile virus.

To date, several investigations have been carried out on the importance of apoptosis in different virus infections, pathogenesis and replication, but many issues are still unclear and under debate (Refs 212, 213, 214). As summarised in Figure 7, a number of arboviruses such as Sindbis virus, *WNV* and *JEV* seem to use apoptosis as a virulence factor to promote their own pathogenesis (215, 216, 217). Each of these viruses has specific targets and biochemical-induced mechanisms during virus-induced programmed cell death. The observations suggest that Sindbis virus-induced apoptosis plays an important role in Sindbis virus pathogenesis and mortality (Ref. 215). After entry of Sindbis virus into the host cell and subsequent formation of Sindbis virus double-stranded RNA intermediates, dsRNA-dependent protein kinase (PKR) recognises these particles (Refs 218, 219, 220). PKR blocks cellular translation through eIF2a phosphorylation, which later can inhibit Mcl-1 (anti-apoptotic Bcl2 family protein) biosynthesis (Ref. 221). PKR also controls c-Jun N-terminal kinases (JNK) through IRS1 phosphorylation and later activates 14-3-3 (Ref. 222). Thus, 14-3-3 affects the accessibility of substrates (e.g., Bad) to kinases and serves to localise kinases to their substrates, thereby leading to release of Bad and disruption of the complex between anti-apoptotic Bcl2 family proteins, Bcl-xl and Bak. Both Bad and Bik can displace Bak from Mcl-1, which results in Bak oligomerization and cytochrome c release, and subsequent induction of apoptosis (Ref. 222). *CHIKV* triggers the apoptosis machinery and uses apoptotic blebs to evade immune responses and facilitate its dissemination by infecting neighboring cells (Ref. 223). *CHIKV* infection can induce apoptotic cell death via at least two apoptotic pathways: the intrinsic pathway, which has been reported to be involved in virus replication and results in activation of caspase-9, and the extracellular pathway, which is dependent on the induction of cell surface or soluble death effector ligands that activate caspase-8. Thus, both pathways activate caspase-3 and finally induce cell death, and this facilitates virus release and spread (Ref. 211). The replication of *Crimean-Congo haemorrhagic fever* virus (*CCHFV*), an arbovirus from the family *Bunyaviridae*, is associated with the death receptor pathway of apoptosis. Up-regulation of pro-apoptotic proteins (i.e. BAX and HRK) and novel components of the ER stress-induced apoptotic pathways (i.e. PUMA and Noxa) have also been shown in a *CCHFV*-infected hepatocyte cell line, which suggests a link between *CCHFV* replication, ER stress and apoptotic pathways. Notably, differential high levels of transcription factors, such as CHOP, which are activated through ER stress, are present in hepatocytes following *CCHFV* replication (Ref. 224). In this study, it was shown that the over-expression of IL-8, an apoptosis inhibitor, during *CCHFV* infection was independent from apoptotic pathways. However, in other studies, a positive correlation was detected between IL-8 induction and *DENV* infection (Refs 224, 225, 226). In contrast to Sindbis virus, *CHIKV* and *CCHFV* replication in infected cells have been proposed to be necessary for apoptosis induction, as demonstrated by the use of UV-inactivated viral particles (Refs 227, 228, 229). The replication of Flaviviruses (e.g. *WNV, JEV* and *DENV*) can be limited by virus-induced programmed cell death at the early stage of virus infection. These viruses might block or delay apoptosis via activating several cell survival pathways, such as PI3K/Akt signalling, to improve their replication rate (Refs 227, 230). Blocking PI3K (using LY294002 and wortmannin) showed that the induction of apoptosis might be a result of p38 MAPK activation and did not affect *JEV* and *DENV* viral particle production (Ref. 227). In 2001, del Carmen Parquet et al. demonstrated that WNV-induced cytopathic effect was caused during induction of apoptosis and that viral replication is an essential event for virus-induced cell death (Ref. 231). *WNV* capsid protein has an anti-apoptotic role, ensuring that it can block or delay apoptosis by suppression of the phosphatidylinositol (PI) 3-kinase-dependent process at the early stage of infection (Ref. 230). In addition, Akt is a downstream target of PI3-kinase and can directly phosphorylate the pro-apoptotic protein Bad at position Ser 136 (Ref. 232). *WNV* can initiate apoptosis through caspases-3 and -12 and p53 after several rounds of replication and it is noteworthy that initial viral dose exerts an influence on kinetics of *WNV*-induced cell death (Refs 228, 233, 234, 235). After some RNA virus infections, expression of multiple miRNAs in host cells might have either a positive or negative effect on virus replication. One such cellular miRNA, Hs_154, limits *WNV* replication by inducing apoptosis through inhibition of two anti-apoptotic proteins like CCCTC binding factor (CTCF) and EGFR-co-amplified and overexpressed protein (ECOP) (Refs 227, 236). *JEV,* an RNA virus, may induce ROS-mediated ASK1-ERK/p38 MAPK activation and thus lead to initiation of apoptosis (Ref. 237). In mouse neuroblastoma cells (line N18) infected with ultraviolet-inactivated *JEV* (UV-JEV), replication-incompetent *JEV* virions induced cell death through a ROS-dependent and NF-kB-mediated pathway (Ref. 238). Initial suppression of UV-JEV-induced cell death, followed by co-infection with active or inactive *JEV*, showed that *JEV* may trigger cell survival signalling to modify the cell environment for timely virus production (Ref. 238). NS1′ protein, a neuroinvasiveness factor that is only produced by the *JEV* serogroup of Flaviviruses during their replication, was introduced as a caspase substrate in virus-induced apoptosis; however, use of a caspase inhibitor had no effect on virus replication (Ref. 239). Empirical evidence showed that *JEV* can affect Bcl-2 expression to increase anti-apoptotic response rather than anti-viral effect to enhance virus persistence and reach equilibrium between replication and cell death (Ref. 240).

Numerous *in vitro* studies have confirmed that *DENV* can induce apoptosis in a wide variety of mammalian cells including endothelial cells, hepatocytes, mast cells, monocytes, dendritic cells and neuroblastoma cells, but the mechanisms are not completely understood. Dendritic cells are believed to be the primary *DENV* targets that play central roles in supporting active replication during virus pathogenesis. However, a recent study reported that *DENV* replication in monocyte-derived dendritic cells (mdDCs) was positively correlated with pro-inflammatory cytokine secretion such as TNFα and apoptosis (Ref. 241). To achieve high replication in macrophages, hepatoma and dendritic cells, *DENV* may subvert apoptosis by inhibiting NF-kB in response to TNFα stimulation (Refs 242, 243). Interaction between *DENV* capsid protein and the hepatoma cell line (Huh7) calcium modulating cyclophilin-binding ligand (CAML) also positively affected viral replication by inhibiting apoptosis (Ref. 243). Activation of p53-dependent apoptosis by *DENV* may also contribute to inhibition of inflammation and reduce immune responses to efficiently disseminate viral progeny (Ref. 244). Microarray analysis following *DENV* infection in p53-positive and -deficient cell lines revealed that activation of the pro-apoptotic gene caspase-1 played a basic role in p53-mediated apoptotic pathway and was necessary for up-regulation of numerous immune response genes (Ref. 244). As mentioned, apoptosis serves as a critical and final step in viral infectious cycles that may favour virus propagation. The pro-apoptotic NSs and anti-apoptotic NSm proteins of the Phelebovirus genus of the family Bunyaviridae (e.g. *RVFV*) delayed apoptosis to efficiently replicate by regulating p53 (Refs 235, 245). The *RVFV* protein inhibits either caspase-8 activity or the death receptor-mediated apoptotic pathway to regulate pro-apoptotic p53 signalling (Ref. 246). NSs can facilitate viral translation through inhibition of PKR/eIF2a pathway and IFN production at early stages of infection (Ref. 247). Members of the Orthobunyavirus genus, family *Bunyaviridae*, delay apoptosis through anti-apoptotic effects of NSs nonstructural protein on IRF-3 activity (Ref. 248).

Apoptosis has also been extensively linked to reovirus replication. *BTV* induces apoptosis in three mammalian cell lines but not in insect cell lines that were tested. *BTV*-mediated apoptosis involved activation of NF-kB and required virus uncoating and exposure to both outer capsid proteins VP2 and VP5 (Ref. 249). Apoptosis was mediated by both intrinsic and caspase-dependent extrinsic pathways (Ref. 250). *African horse sickness virus* (*AHSV*), another orbivirus, also induced apoptosis in mammalian BHK-21 cells but not in insect KC cells, through activation of caspase-3 (Ref. 251).

When apoptotic programmed cell death acts as a barrier against viral replication, previous research has revealed that some arboviruses can delay or block apoptosis to elevate their replication and dissemination. Moreover, viral replication of some arboviruses occurs following the presence of viral-induced apoptosis. However, the exact mechanisms whereby viruses modulate apoptosis in different mammalian cells need to be more extensively studied.

## Arboviruses and UPR

The scientific literature related to the role of UPR in arbovirus pathogenesis is limited. Here, we review some of the arboviruses and the UPR pathways they elicit to aid replication. *WNV* is a neurotropic arbovirus that emerged as a pathogen of serious concern in the North American population. People infected with *WNV* are affected by severe neurological diseases such as meningitis, encephalitis and poliomyelitis (Ref. 233). *WNV* activates multiple UPR pathways leading to transcriptional and translational activation of several UPR target genes (Ref. 233). Of the three UPR pathways, the XBP1 pathway was shown to be non-essential for *WNV* replication and it was replaced by other pathways. ATF6 was degraded by the proteasome and PERK transiently phosphorylated eIF2α and induced the pro-apoptotic protein CHOP (Ref. 233). *WNV*-infected cells showed signs of apoptotic cell death including induction of growth arrest, activation of caspase-3 and activation of poly (ADPribose) polymerase (PARP). *WNV* titer levels were also significantly increased when grown in a CHOP^−/−^ deficient mouse embryo fibroblast (MEF) cell line but not in wild type MEF cells (Ref. 233). This evidence showed that *WNV* activates the UPR, and a host mechanism to counteract *WNV* infection involved activation of CHOP-dependent cell death (Ref. 233). In another study, the *WNV* Kunjin strain activated UPR signalling upon infection in mammalian cells (Ref. 252). UPR ATF6/IRE1 pathways were activated by this strain. However, there was no significant phosphorylation of eIF2α indicating that the UPR PERK pathway was not activated (Ref. 252). The Kunjin strain nonstructural proteins, NS4A and NA4B, were potent inducers of UPR. Moreover, sequential removal of NS4A hydrophobic domains decreased UPR activation but increased interferon gamma-mediated signalling (Ref. 252). These results show that *WNV* Kunjin strain activates UPR signalling and hydrophobic residues of WNV nonstructural proteins regulate the UPR signalling cascade. The role of ATF6 signalling in *WNV* replication is poorly understood. Results from the same group showed that ATF6 signalling is required for *WNV* replication by promoting cell survival and inhibition of the innate immune response (Ref. 253). ATF6-deficient cells showed a decrease in protein and virion production when infected with *WNV* Kunjin strain. These cells also demonstrated increased eIF2a phosphorylation and CHOP transcription, but these events were absent in infected control cells (Ref. 253). In contrast, IRE I-deficient cells do not show any discernible differences when compared with IRE I-positive cells upon infection (Ref. 253). These results also demonstrate that, in the absence of ATF6, other UPR signalling cascades such as PERK and IRE1 pathways cannot activate or enhance virus production, indicating that ATF6 is required for viral replication. However, it has also been shown that both ATF6 and IRE I are required for signal transducer and activator of transcription (STAT) I phosphorylation, showing that ATF6 is required for inhibition of innate immune response (Ref. 253). The arboviruses *CHIKV* and Sindbis also cause frequent epidemics of febrile illness and long-term arthralgic sequelae that affect the lives of millions of people each year (Ref. 254). These viruses replicate in infected patients and also in mammalian cells indicating that they have certain control over the UPR of the host system. Analysis of these viral infections in mammalian cells shows that *CHIKV* specifically activates the ATF-6 and IRE-1 branches of the UPR pathway and suppresses the PERK pathway (Ref. 254). *CHIKV* nonstructural protein 4 (nsp4) expression in mammalian cells suppresses eIF2α phosphorylation that regulates the PERK pathway (Ref. 254). These results provide insight on the replication of *CHIKV* in mammalian cells by regulating the host UPR mechanism. However, experimental findings with Sindbis virus show that it induced uncontrolled UPR, which is reflected by failure to induce synthesis of ER chaperones, followed by increased phosphorylation of eIF2α and activation of CHOP leading to premature cell death (Ref. 254). In another study, it was reported that the UPR XBP1 pathway was activated when neuoroblastoma N18 cells were infected with the arboviruses *JEV* and *DENV* (Ref. 255). This was evidenced by splicing of XBP1 mRNA and activation of downstream genes *ERDJ4, EDEM1* and *p58*. Reduction of XBP1 by small interfering RNA had no effect on cellular susceptibility to the two viruses but enhanced cellular apoptosis (Ref. 255). Overall, these results suggest that both encephalitis and *DENV* trigger the XBPI signalling pathway and take advantage of this cellular response to alleviate virus induced cytotoxicity (Ref. 255). According to another group, *DENV* infection of A547 ovarian cancer cells elicited the UPR signalling response (Ref. 256). This was demonstrated by phosphorylation of eIF2α. It was also shown that different serotypes of *DENV*, such as ATF6 and IRE1, activate other UPR pathways. These results show that different *DENV* serotypes have the capacity to modulate different UPR pathways. They also demonstrated that de-phosphorylation of eIF2α by a drug called solubrinal reduced virus infection. This unique report showed that the same virus could activate all three UPR pathways (Refs 256, 257).

Initiation of UPR signalling is critical for cell survival and also for viral replication. All the above results show that arboviruses induce UPR signalling upon infection in mammalian cells. However, the UPR pathways that are activated upon infection with various arboviruses are not the same. Even different strains of the same virus activate different UPR pathways. These results suggest that specific virus-induced UPR pathway usage depends on the type of viral strain used. In vitro studies using ectopically-expressed arbovirus nonstructural proteins alone in mammalian cells showed that the proteins themselves can elicit the UPR response. Mutations of certain hydrophobic residues in nonstructural proteins reduced the UPR signalling response. These results indicate that composition of viral nonstructural proteins can determine the type of UPR pathway to be elicited and the extent of UPR response. Viral nonstructural proteins often undergo mutation; thus, more studies are needed to understand the role of arbovirus nonstructural proteins in inducing UPR. The role these viruses may play in UPR in the invertebrate insect cells is even less defined. Thus, induction of UPR signalling by viruses is one important facet, and equally important is how these viruses respond to anti-viral therapy. Do these viruses use the UPR pathways to decrease the effectiveness of anti-viral therapies? This is also one of the main questions to be answered. Thus, in conclusion, a significant amount of research is needed to investigate the pathogenesis of arboviruses and their relationship with UPR signalling. These studies can provide us with better antiviral therapeutics to control arbovirus replication by addressing various mechanisms of virus propagation.

## Conclusion

Arbovirus infections lead to serious health issues in many parts of the world. To date, there is no treatment for most arbovirus infections and vaccines have been recently developed for only a few of these arboviruses. Therefore, finding a way to increase the efficiency of current therapeutic approaches to arbovirus infections will improve health conditions in many areas of the world. As has been discussed in this review, arbovirus infection can stimulate apoptosis, autophagy, or UPR in infected cells or organs. Activation of these pathways usually interferes with arbovirus replication and infection processes. Therefore, modulating these pathways may be a part of future strategies to combat arbovirus infections.

Apoptosis, autophagy and UPR have been widely investigated in many diseases including cancer, cardiovascular diseases and pulmonary diseases. Many inhibitors and inducers of these pathways have been developed to improve treatment protocols in these diseases. Since apoptosis, autophagy and UPR are tightly interconnected with each other and usually affect each other, it is critical to find out which pathway is the dominant one in the arbovirus infection process and how it regulates viral infection and replication in the infected cells. It is very important to identify the extent of apoptosis, autophagy, and UPR alterations in virus infected cells. After identifying these changes it would be very important to address how induction/inhibition of these pathways would modulate virus replication, and production of active viral particle in infected cells. As an example, we can modulate UPR using inducers (thapsigargin) or inhibitors (PERK GSK inhibitor, IRE1 inhibitor) and find out how these treatments effect arbovirus replication. These findings would provide better opportunities to use the modulation of these pathways for better designing therapeutic strategies and controlling viral infection. If this question can be clearly answered, induction or inhibition of these pathways may represent a novel enhanced treatment or prevention strategy against arbovirus infections.
